# Supplementary material for: MetaRibo-Seq measures translation in microbiomes
Source: Nat Commun. 2020 Jun 29;11:3268. doi: 10.1038/s41467-020-17081-z (PMC7324362; doi:10.1038/s41467-020-17081-z)
Supplement: Supplementary file 10 — Supplementary Data 7 [file 41467_2020_17081_MOESM10_ESM.zip › File2/Confidence_High_Taxonomy/402359_out.krona.html]

Javascript must be enabled to view this page.

members
magnitude
magnitudeUnassigned
count
unassigned
taxon
rank

402359\_out

61

superkingdom
2
61

phylum
61
1239

61
186801
class

order
61
186802

family
186803
61

genus
841
61

360807
61
species

SRS011084\_contig\_number\_32828SRS011405\_contig\_number\_contig-100\_52.108957SRS012849\_contig\_number\_8661SRS013951\_contig\_number\_15571SRS014459\_contig\_number\_36868SRS014923\_contig\_number\_35788SRS015065\_contig\_number\_16935SRS015431\_contig\_number\_71021SRS015578\_contig\_number\_32935SRS015663\_contig\_number\_18225SRS015890\_contig\_number\_22169SRS016335\_contig\_number\_26068SRS016954\_contig\_number\_27470SRS017433\_contig\_number\_12099SRS018888\_contig\_number\_3497SRS018984\_contig\_number\_31779SRS019068\_contig\_number\_35632SRS019161\_contig\_number\_38552SRS019381\_contig\_number\_19569SRS019397\_contig\_number\_1940SRS019910\_contig\_number\_10419SRS023346\_contig\_number\_8194SRS048164\_contig\_number\_27724SRS049959\_contig\_number\_48231SRS050998\_contig\_number\_15216SRS051031\_contig\_number\_17817SRS052027\_contig\_number\_12858SRS058070\_contig\_number\_11479SRS058770\_contig\_number\_29134SRS063518\_contig\_number\_25243SRS064276\_contig\_number\_29050SRS065504\_contig\_number\_28481SRS075021\_contig\_number\_7019SRS076804\_contig\_number\_319SRS097889\_contig\_number\_contig-100\_254.227908SRS097920\_contig\_number\_28844SRS098827\_contig\_number\_6224SRS103987\_contig\_number\_18234SRS104693\_contig\_number\_9518SRS105153\_contig\_number\_38820SRS142712\_contig\_number\_3959SRS143085\_contig\_number\_3117SRS143148\_contig\_number\_26920SRS143417\_contig\_number\_4920SRS143722\_contig\_number\_15989SRS143780\_contig\_number\_22466SRS144135\_contig\_number\_15658SRS144297\_contig\_number\_7340SRS144362\_contig\_number\_42187SRS144506\_contig\_number\_48377SRS144714\_contig\_number\_contig-100\_497.172879SRS146764\_contig\_number\_31915SRS147271\_contig\_number\_37635SRS147977\_contig\_number\_12366SRS148091\_contig\_number\_13074SRS148159\_contig\_number\_7231SRS148253\_contig\_number\_13061SRS148784\_contig\_number\_14536SRS893288\_contig\_number\_2421SRS893327\_contig\_number\_11327SRS893342\_contig\_number\_4754
